# Group Membership and Social Identities in a Formative Intervention in a Mexican Hospital

**DOI:** 10.3389/fpsyg.2021.786054

**Published:** 2021-12-09

**Authors:** Hugo Armando Brito Rivera, Francesca Alby, Cristina Zucchermaglio

**Affiliations:** ^1^Independent Researcher, Mexico City, Mexico; ^2^Department of Social and Developmental Psychology, Sapienza University of Rome, Rome, Italy

**Keywords:** social identity, formative intervention, collective pronouns, group membership categorization, rhetorical resources

## Abstract

Formative intervention is a participatory methodology that supports organizational change by means of an interactive and systematic dialogue carried out by researchers and participants. In this process, the researchers contribute to expanding the conversational space in the organization by supporting participants in examining and reflecting on their own work practices, as well as in modeling, shaping, and experimenting with innovations. Drawing on transcripts of videotaped sessions, this study analyzes how change is discursively sustained by the researchers who conduct the meetings within a formative intervention in a Mexican hospital. The quantitative and qualitative analysis focuses on the collective pronoun “we” as a membership categorization device deployed by the researchers for rhetorical and pragmatical aims, such as questioning about the state of necessity for the intervention, engaging the participants, or introducing a proposal of innovation with the participants. Results show how group membership and social identity markers are used by researchers to support emerging forms of collaboration, involvement of participants and the creation of common ground during the intervention process. In terms of the practical implications of the study, an informed and strategic use of membership categorization devices used by the researcher can increase the effectiveness of their formative and expansive role.

## Introduction

### Formative Interventions for the Development of Workgroups

Formative intervention is a participatory methodology to support a group of professionals, within a work activity system, in the analysis, design, and implementation of new ways of working (Engeström, 2011). In this model, innovations are negotiated with the practitioners, and data are collected to explore the functioning and potential of such groups in their own context. The process of qualitative improvement and change pursued by this methodology is conceived as a progressive increase in the co-construction of new meanings and new work practices (Zucchermaglio and Alby, 2006). Change is approached as an emergent property of the participating organizational group, and the competencies that are needed to develop such a process are considered as located and distributed within it.

The methodology of formative intervention is implemented and developed, in practice, by means of “a toolkit called the Change Laboratory” (CL) (Engeström, 2015), inspired by the work of Vygotskij (1934/1990) and based on the activity theory (Engeström et al., 1996; Virkkunen and Newnham, 2013). This methodology has been widely applied in Europe (see Engeström, 2005), both in healthcare and educational organizations (Stoppini et al., 2009; Ivaldi and Scaratti, 2020) and is at an early stage of development in Latin America (see Montoro and Brito, 2017; Pereira-Querol et al., 2019; Brito, 2020; Vilela et al., 2020).

The CL, which is essentially based on the guided activation of “self-managed” improvement processes, consists of a series of sessions in which practitioners from an organization are supported by means of a bottom-up participatory approach in analyzing the history of their activity system, including sociohistorical tensions and contradictions. One of the main objectives of a CL process consists of unlocking the transformative agency of practitioners to become protagonists in the creation and implementation of a new model of practice (Engeström, 2015; Laitinen et al., 2016). The CL methodology does not provide prepackaged solutions “from the outside” but aims to bring out and develop in the participating group the ability to act in order to transform the activity system in which they work.

To this end, participants are actively and directly involved in the analysis, sensemaking process, and interpretation of their own activity relying on the data collected by means of individual and group interviews, microanalysis of significant and/or problematic observations, narratives, and/or video interactions (Ivaldi and Scaratti, 2020). Such a process involves several sessions and might lead to finding new solutions to problems and possibilities for organizational “expansion.” This type of intervention requires the contribution and experimental attitude of the researchers who play an active role that goes beyond the traditional perspective of a casual observer and facilitator (Engeström, 2015). In this sense, for participants to take the lead in the design and experimentation of new working practices, researchers introduce their own ideas and intentions with the aim of provoking and sustaining a cycle of expansive learning.

The role of the researcher is, therefore, to expand the conversational space during the intervention in order to support participants in examining and reflecting on the current state of their activity system, as well as in modeling, shaping, and experimenting with innovations. Researchers have to sustain dialogic sensemaking when creating socially useful knowledge, acting based on a sort of anticipational fluidity (Cunliffe and Locke, 2020) as a way of relating with others when working with differences. Such differences are experienced through the unfolding living flow within the moment of conversation.

The contribution of researchers is based on the collaborative introduction and application of new tools and ideas through “determined and systematic dialogue” (Engeström, 2015). Consequently, the dynamism of the intervention stems from the interplay of ideas and intentions between researchers and participants. During a CL, sessions are videotaped for the collection of longitudinal data to analyze the intervention process and its development, including the interactions between researchers and the participating group.

In this study, the data collected from the videotaped sessions are employed to examine the discursive practices of researchers during the intervention.

### Social Identity as a Rhetorical and Pragmatic Resource

Studies carried out on intergroup relations in theories of social identity (Tajfel, 1981) and self-categorization (Turner et al., 1987) have constituted one of the richest areas of experimental social psychology. Tajfel’s theory explains how social identity influences intergroup behavior, whereas Turner’s theory of self-categorization focuses on the psychological nature of belonging to a group and on the socio-psychological basis of group behavior. Most of the empirical research carried out in this area has concentrated on intergroup behavior between large social groups (distinguished by race, nation, ethnic group, and so forth) or in experimentally created groups (using the technique of minimal paradigms; Billig and Tajfel, 1973). But what happens in small groups, characterized by multiparty interactions between members? For discursive social psychology (Billig, 1987; Cole, 1996; Edwards, 1998), social (and self) categorization processes are situated results of negotiation discursive practices occurring in interactions with others. In this view, identity is “something that people do which is embedded in some other social activity, not something they are” (Widdicombe, 1998:191).

Sacks (1992) highlighted how social identity choices and moves are both indexical (defined by the terms used to mark the belonging social categories to give salience to) and occasioned (there is a particular social context where the categories should assume some relevance). Each group member has different identities to show and to give salience to for positioning the self and the other rhetorically (Hester and Eglin, 1997). The choice between these possibilities of positioning the self and the others (Harré, 1989; Muhlhausler and Harré, 1990) is guided by social factors such as the relationship between member, their roles, and the content and scope of the group. Identity is a resource that participants are using rhetorically and strategically during social interactions, but the possibility of using different social identities to negotiate and build ingroup-outgroup categorizations is also context-related (Zucchermaglio, 2005; Fantasia et al., 2021). Social identities can become an important negotiation content between members of a group rather than a stable characteristic and an *a priori* of discourse in interaction. The social context itself plays an active role in allowing some possible identity choices but also in defining the access of each group member to the identity negotiation process.

Many studies have shown how, even using minimal lexical choices, speakers mark those aspects of their own (or other) social identity that they intend to present as relevant in specific interactive contexts (Drew and Heritage, 1992; Sacks, 1992; Hester and Eglin, 1997). In particular, pronouns are discursive components that, together with lexical selection (Drew, 1992), could act as a powerful “membership categorization device.”

In this theoretical and methodological framework, we focus on how the researcher, as part of an intervention process, discursively supports change in the group of healthcare professionals of a Mexican hospital, through the construction and negotiation of specific social identities. Specifically, we present both quantitative analysis and qualitative analysis of how, among the various discursive devices at hand, the researcher strategically uses the pronominal markers “we” to evoke social groups and identities that are rhetorically functional to achieve the goals of the organizational change intervention. Our aim is to provide a micro- and discursive analysis of the strength and effectiveness of organizational change interventions, usually and mostly described and analyzed by considering more general dimensions of participation.

## Methods

This study is based on a formative intervention carried out during May and June 2017 in a public hospital located in the central part of Mexico (State of Guanajuato). The intervention was conducted by a multidisciplinary team of 2 senior researchers and 2 assistants with the participation of 25 members of the hospital board of directors. The intervention was requested to support the participants in the analysis of their work activity and functioning as a professional team.

The group of participants consisted of area coordinators, heads of departments, and hospital directors, both medical and administrative. The intervention was conducted through seven sessions, with an average duration of 80 min and at a rate of one per week. The language used in the sessions was Spanish. The objectives of the intervention were to increase integration, collaborative work, and improve interprofessional relations, as a starting point to enhance the performance and effectiveness of the board of directors (Montoro and Brito, 2017). As an “external” measure of the achievement of these objectives (which is not the focus of the paperwork), a questionnaire was distributed to all participants at the end of the last work session. Analysis of the responses reveals relevant clues of agentive speech in the participants and a greater willingness to engage collaboratively with coworkers.

The data analyzed in the study are the verbatim transcripts of the seven intervention’s videotaped sessions. As a preliminary analysis, we first read through the transcripts to explore the presence and distribution of pronouns in the participants’ discursive sequences. Since pronouns are easily identifiable in transcriptions, their occurrence constitutes a finite class that can be identified and counted. We counted, therefore, occurrences of “we” (“nosotros” in Spanish) in each session. Moreover, we also highlighted who was the speaker and which collective identity categories were marked in each session.

As pronouns in Spanish are implicitly marked by verbs that vary according to the grammatical person, their marking in discourse is not necessary: I, you, we, and so forth can, and often are, be elided as they are implicitly marked by the verbs according to the grammatical person. In other words, their occurrence in conversation requires motivation and has a rhetorical function. For this reason, the use of the collective pronoun “we” acquires a specific relevance, as it represents a particular choice of the speaker. The quantitative analysis oriented the subsequent qualitative analysis and codification, which focused on how, when, and with what rhetorical functions the researchers used collective identity categorizations in the sessions. Subsequently, a micro- and discursive analysis focused on the identities marked during the first and fourth sessions was carried out to discuss exemplary excerpts corresponding to each identity category and rhetorical function.

## Results

### Preliminary Analysis of Identity Category Occurrences

Our preliminary analysis on the occurrences of the “we” pronoun was functional for identifying the identity categories that emerged and how often these categories were employed by participants. Table 1 provides a quantitative overview of the identity categories that emerged across the sessions and of the speakers marking them. Among the participants, the researcher results to be the one who most frequently invokes collective identities in every session (except for session n. 3, see Table 1).

**TABLE 1:** Identity categories marked by the researchers during the intervention.

Session	N. “we”	N. “we” marked by researchers	N. identities marked by researchers	Identities categories marked by researchers
1	40	29 (72.5%)	4	Board of Directors Incumbent Researchers Researchers in the room All the people in the room
2	57	37 (65%)	2	Incumbent Researchers All the people in the room
3	37	12 (32.3%)	2	Board of Directors Incumbent Researchers
4	48	35 (72.6%)	3	Board of Directors Incumbent Researchers All the people in the room
5	12	7 (58%)	2	Incumbent Researchers All the people in the room
6	13	7 (54%)	2	Incumbent Researchers All the people in the room
7	32	24 (75%)	3	Incumbent Researchers Researchers in the room Group of participants in the shadowing technique

The identity categories that were codified from the data and account for the “we” marked by the researchers during the sessions are as follows:

*Board of directors*. This “we” was codified each time the researcher referred to the group of the board of directors, i.e., area coordinators, heads of department, and hospital directors, both medical and administrative, who could be in the room during the session or not.

*Incumbent researchers*. This “we” was codified when the researchers referred to themselves as senior researchers in charge of the intervention and responsible for conducting the sessions.

*Researchers in the room*. This code states the “we” used to point out the research team in a broad sense. It was codified each time the researcher indicated the research team made up of two incumbent researchers and two junior researchers, who were present in the session and jointly were in charge of the setting organization, video cameras placement, preparation of material, and so forth.

*All the people in the room*. This “we” was codified each time the researcher referred to all the participants present in the session, both members of the board of directors, senior researchers, and assistants.

*Participants in the shadowing technique*. This group was codified when the researcher pointed out to the participants in a shadowing exercise carried out in the last two sessions (codified just once in session n. 7).

The identity categories made relevant by researchers appeared as broadly linked to the aims of the different sessions of the intervention. We focused here on session n. 1 (Table 2) and session n. 4 (Table 3) since they were particularly central for the successful accomplishment of this intervention. The specific objectives of the first session were to question the state of necessity for the intervention as well as to engage the participants. In the fourth session, the aims were centered on supporting the participants in focusing on improvement areas and proposing alternatives and innovations to improve their work practices. In the following paragraphs, we described and provided examples of how identity categories were used by researchers in these sessions.

**TABLE 2:** Exemplary excerpts of identity categories used by researchers in the first session.

Spanish	English
Función retórica: *Para describir y contextualizar lo que hacen los investigadores de acuerdo al modelo de intervención.*	Rhetorical function: *To describe and contextualize what researchers do according to the intervention model*.
Extracto 1. Grupo evocado: Investigadores titulares	Excerpt 1. Evoked group: Incumbent researchers
*Investigador A*: (…) >cómo trabajamos?, de abajo hacia arriba, **nosotros** no llegamos de arriba como universidad con el conocimiento hacia abajo, a impartir nada, sino que **nosotros** tenemos micrófonos, equipo de grabación, porque todo lo que se va a platicar aquí, se va a analizar, los problemas y las soluciones van a surgir de lo que ustedes digan, no de lo que **nosotros** pensemos o digamos de antemano, al entrar aquí, tenemos que olvidar **nosotros** lo que sabemos, que no sirve para nada, **nosotros** venimos a escucharles a ustedes, sobre todo la solución va a surgir de ustedes mismos (…)	*Researcher A*: (…) How do we work? from the bottom up, **we** do not come from above as a university with knowledge downward, to impart anything, but **we** have microphones, recording equipment, because everything that is going to be discussed here, is going to be analyzed, the problems and solutions are going to arise from what you say, not from what **we** think or say beforehand, by entering here, we have to forget what **we** know, which is useless, **we** come to listen to you, above all the solution is going to arise from you yourselves (…)
Extracto 2. Grupo evocado: Investigadores en la sala	Excerpt 2. Evoked group: Researchers in the room
*Investigador A*: (…) comunidad es lo que en inglés llaman *stakeholders* que son todas las partes interesadas (…) todo aquel que tenga un interés en la actividad que ustedes hacen. Incluso **nosotros**, **nosotros** somos parte ahora de la comunidad, porque nos interesa lo que hacen ustedes, queremos aprender y descubrir, la comunidad es muy amplia y es la base de la actividad (…)	*Researcher A*: (…) community is what in English they call *stakeholders*, which are all the interested parties (…) everyone who has an interest in the activity you do. Even **us**, **we** are now part of the community, because we are interested in what you do, we want to learn and discover, the community is very broad and is the basis of the activity (…)
Función retórica: *Para apoyar y sostener la participación / reflexión*	Rhetorical function: *To support and sustain participation / reflection.*
Extracto 3. Grupo evocado: Cuerpo de Gobierno	Excerpt 3. Evoked group: Board of Directors
*Investigador A*: (…) entonces me hablan de mil cabezas, de brazos, yo veo que este es un cuerpo, yo digo pues ya casi tengo aquí el ser entero >no?, yo les pregunto si **nosotros** somos el cuerpo, luego está el IMSS que tiene mil cabezas, los operativos parece que son las extremidades, los brazos, las piernas, >si será esa metáfora adecuada para tratar de entender lo que sucede aquí? >Qué opinan ustedes? (…) >qué significa eso de que el IMSS es un monstruo de mil cabezas?	*Researcher A*: (…) so they talk to me about a thousand heads, about arms, I see that this is a body (Body means “cuerpo.” In this excerpt, when talking about “body,” participants are talking about two meanings interrelated: “cuerpo” and “Cuerpo de Gobierno,” but in English such connotation cannot be observed in the translation of the term “Cuerpo de Gobierno” as “board of directors.”), I say well, I almost have the whole being here, don’t I, I ask you, if **we** are the body, then there is the IMSS (IMSS: Mexican Social Security Institute for its acronym in Spanish that has a thousand heads, the workers seem to be the extremities, the arms, the legs, is that an appropriate metaphor to try to understand what is happening here? What do you think? (…) What does it mean to say that IMSS is a monster with a thousand heads?
*Director del hospital*: El expresar que el instituto haciendo una analogía con un monstruo de mil cabezas es hablar de que es una institución sumamente compleja que tiene no sólo un pilar en el cual se basa la atención médica sino que también tiene atención a prestaciones económicas y sociales, que lo sabemos también es un rubro muy importante dentro del instituto y que por lo tanto la diversidad de áreas que interactúan para que se lleven a cabo los objetivos para los cuales fue creada esta institución lo hacen tan complejo como un monstruo de mil cabezas (…)	*Hospital director*: When expressing that the institute, drawing an analogy, is like a thousand-headed monster is to say that it is an extremely complex institution that has not only a pillar on which medical care is based but also economic and social benefits, which we know is also a very important area within the institute, and therefore the diversity of areas that interact to achieve the objectives for which this institution was created make it as complex as a thousand-headed monster (…)
Extracto 4. Grupo evocado: Grupo de los presentes	Excerpt 4. Evoked group: People in the room
*Investigador A*: Supervisión, vinculación, dirección, el modelo habla de control, esas son actividades que ustedes hacen, pero el título de la película, me están diciendo, hay un escena de acción, una escena romántica, una de guerra, pero, >el nombre de la película?, el modelo dice que la función o lo que ustedes hacen como Cuerpo de Gobierno se llama gestión de la unidad médica, gestión o gestión directiva del hospital, esa es su función según el modelo, >se sienten cómodos con esa definición?, >o la cambiamos, la modificamos?, es nuestra, **nosotros** la podemos hacer como **nosotros** queramos, como nos sintamos cómodos, pero sí es importante que le pongamos nombre porque si no es el monstruo de mil cabezas, da miedo, no sé exactamente, algo hacemos, un poco de esto un poco de aquello, > gestión?	*Researcher A*: Supervision, liaison, direction, the model talks about control, these are activities that you do, but the title of the film, you are telling me, there is an action scene, a romantic scene, a war scene, but what is the name of the film?, the model says that the function or what you do as Board of Directors is called management of the medical unit, management or management of the hospital, that is your function according to the model, do you feel comfortable with that definition, or do we change it, modify it?, it is ours, **we** can do it as **we** wish, as we feel comfortable, but it is important that we give it a name because otherwise it is a monster with a thousand heads, it is scary, I don’t know exactly, we do something, a bit of this, a bit of that, management?
*Jefa de finanzas*: Gestión, gestionamos, finalmente.	*Head of finance*: Management, we manage, finally.
*Investigador A*: La gestión abarca todo, >no?	*Researcher A*: Management is all-encompassing, isn’t it?
*Director del hospital*: Si partimos de la base de que gestionar es que las cosas sucedan, pues sí, porque cada quien en su ámbito de competencia hace eso, gestión.	*Hospital director*: If we start from the premise that management means making things happen, then yes, because everyone in their area of competence does that, management.

*The correspondence between the category identities marked in Spanish and its respective equivalency in English (“nosotros” and “we/us,” respectively) are indicated in bold letters.*

**TABLE 3:** Exemplary excerpts of identity categories used by researchers in the fourth session.

Spanish	English
Función retórica: *Para encuadrar el focus de la intervención*	Rhetorical function: *To frame the focus of the intervention*
Extracto 5. Grupo evocado: Investigadores titulares	Excerpt 5. Evoked group: Incumbent Researchers
*Investigador B*: (…) queremos empezar con una breve recapitulación de cómo estamos viendo **nosotros** el hospital y su sistema que implica muchas personas, este es un esquema que C (investigador A) les había compartido desde la primer sesión, aquí **nosotros** creemos que el potencial es ver el hospital como un todo, no es responsabilidad solamente de una persona, el hospital tiene su propia historia, es una actividad colectiva que sale gracias a la implicación de todos, es una red de relaciones entre áreas (…), bueno, lo que a **nosotros** nos gustaría empezar a trabajar, a partir de esta sesión, es cómo desarrollar al Cuerpo de Gobierno (…) **nosotros** queremos ponerles a ustedes en la parte protagónica como Cuerpo de Gobierno y conectar su desempeño con los resultados que obtienen como Cuerpo de Gobierno, para pensar y reflexionar hoy sobre qué herramientas o dónde podríamos ubicar las dimensiones de mejora (…)	*Researcher B*: (…) we want to start with a brief recapitulation of how **we** are seeing the hospital and its system that involves many people, this is a scheme that C (researcher A) had shared with you from the first session, here **we** believe that the potential is to approach the hospital as a whole, it is not only the responsibility of one person, the hospital has its own history, it is a collective activity that comes out thanks to the involvement of everyone, it is a network of relationships between areas (….), well, what **we** would like to start working on, starting with this session, is how to develop the Board of Directors (…) **we** want to put you in the leading role as Board of Directors and connect your performance with the results you obtain as Board of Directors, to think and reflect today on what tools or where we could place the dimensions of improvement (…).
Función retórica: *Para proponer recursos conducentes al cambio*	Rhetorical function: *To propose resources conducive to change.*
Extracto 6. Grupo evocado: Investigadores titulares	Excerpt 6. Evoked group: Incumbent researchers
*Investigador B*: No habiendo alguna más, me gustaría adelantar el siguiente paso, a **nosotros** nos gustaría, sería que de estas cuatro (áreas de mejora) que escuchamos ahorita o si llegáramos a identificar alguna otra, pudiéramos focalizarla y empezar a trabajar en ella (…) eso significaría poner a prueba un pequeño cambio pero antes de ese pequeño cambio pues podemos avanzar en la construcción del problema identificando, y si ustedes están de acuerdo, fijar alguno de estos cuatro, cuál es el que queremos mejorar, pensando en que vamos a obtener otro resultado, ponerlo a prueba y hablarlo después, esto es un modelo que **nosotros** les proponemos, digamos para ganar control sobre el cambio que se quiere hacer y al ganar control en este cambio y ponerlo y evaluarlo, avanzar en lo que se quiere, esto mismo que es digamos un, ciertamente un modelo de gestión relativamente alternativo que **nosotros** les estamos proponiendo desde ahorita, **nosotros** que les parece si después analizamos sus hojas, sus testimonios, les proponemos un área y lo ponemos a prueba (…)	*Researcher B*: Not having any more, I would like to advance the next step, **we** would like, would be that from these four (areas of improvement) that we heard now or if we were to identify any other, we could focus on it and start working on it (….) that would mean testing a small change, but before that small change, we can go ahead in the construction of the problem by identifying, and if you agree, to fix one of these four, which is the one we want to improve, thinking that we are going to obtain another result, test it and talk about it afterward, this is a model that **we** are proposing to you, let’s say to gain control over the change that is wanted, and by gaining control over this change and putting it into practice and assessing it, to advance in what is wanted, this is certainly a relatively alternative management model that **we** are proposing right now, what do you think if **we** analyse your sheets, your testimonies, we propose you an area and we test it (…)
Función retórica: *Para apoyar y sostener la participación / reflexión.*	Rhetorical function: *To support and sustain participation / reflection.*
Extracto 7. Grupo evocado: Cuerpo de Gobierno	Excerpt 7. Evoked group: Board of Directors
*Investigador A*: (…) quisiera saber >qué tanto se involucra a la comunidad?, >qué tanto están en contacto con reuniones con el Gobierno local de Z (nombre de una ciudad)? >Eso está sucediendo?	*Researcher A*: (…) I would like to know how much the community is involved, how much you are in contact with meetings with the local government of Z (name of a city) Is that happening?
*Director del Hospital*: Mes con mes hay un comité municipal de salud, participa en representación la doctora M o va S, va la doctora V, la doctora O, tenemos aparte una vinculación con el comité municipal de vacunación (…) son alrededor de cinco comités en donde se involucra la intervención del municipio, instituciones de salud.	*Hospital Director*: Every month there is a municipal health committee, Doctor M participates on behalf of Doctor S, or Doctor V goes to, or Doctor O, we have apart a link with the municipal vaccination committee (…) there are around five committees where the intervention of the municipality and health institutions is involved.
*Investigador A*: >Cómo se podría involucrar más? (…) allá en la escuela a veces (…) se invitan personas externas para que vengan a platicar de algo (…) >se imaginan ustedes a un paciente aquí?, unos dicen que sí, a otros les da risa nerviosa, >por qué no?, traer un paciente, que el paciente nos cuente de un caso, **nosotros** veamos desde su punto de vista.	*Researcher A*: How could it get more involved? (…) there in the school sometimes (…) people from outside are invited to come and talk about something (…) Can you imagine a patient here?, some say yes, others laugh nervously, why not, bring a patient, let the patient tell us about a case, that **we** see from his point of view?
*Jefa de Trabajo Social*: De hecho, C (nombre del investigador 1), ya en una de las sesiones me di la oportunidad de invitar a un paciente que recibió un tratamiento de un trasplante de riñón (…) ojalá en lo sucesivo esta parte que creo si es bien importante la consideráramos.	*Head of Social Work*: In fact, C (name of researcher A), in one of the sessions I had the opportunity to invite a patient who received a kidney transplant treatment (…) I hope that in the future we will consider this part, which I think is very important.
*Investigador A*: Sí, en este método lo que llamamos es “romper las barreras,” cruzar las barreras la barrera entre **nosotros** y ellos, esa barrera también les dificulta >no?	*Researcher A*: Yes, in this method what we call “breaking down the barriers”, crossing the barriers, the barrier between **us** and them, that barrier also makes it difficult for them, doesn’t it?
Extracto 8. Grupo evocado: Grupo de los presentes	Excerpt 8. Evoked group: People in the room
*Investigador A***:** (…) >Cómo liderar?, pues como nos gustaría que nos lideren a **nosotros**, pongámonos del otro lado, a mí me gusta cuando me tratan así, a mí me gusta cuando me hablan así (…)	*Researcher A*: (…) How to lead, as we would like than someone lead to **us**, let’s get on the other side, I like it when they treat me like that, I like it when they talk to me like that (…)

*The correspondence between the category identities marked in Spanish and its respective equivalency in English (“nosotros” and “we,” respectively) are indicated in bold letters.*

### Identity Categories Used by Researchers in the First Session

During this meeting, the researchers presented the methodology of the intervention, describing and contextualizing its theoretical and epistemological principles. This was the first time they met with the participants and was the initial opportunity to negotiate and inquire them about the state of necessity for the intervention, as required in the formative intervention approach (Engeström et al., 1996).

Excerpt 1 (Table 2) provides an early example of identity categorization employed by researchers, where the “we” indicates the researchers conducting the intervention. There were four researchers in the room but two of them were juniors who observe and do not intervene in the session. Here the “we” refers to senior researchers who represent a university role and methodological expertise (how do we work?). This “we” is fielded to be depowered with respect to the “you” of the participants, who are presented as repositories of knowledge and agency. There is a reformulation of who has the direction of the intervention, from the researchers to the participants, therefore an attempt to propose this role to the participants in order to nominate them to an active and agentic role. The rhetorical intention is, therefore, to explain the intervention methodological approach and describe what researchers do with an informative purpose, but there is also a subtle attempt to persuade the participants by requesting them to get involved in the enterprise and to be its protagonists. Without this adhesion, in fact, the formative intervention model could not have been put into practice according to its main theoretical and methodological principles (Virkkunen and Newnham, 2013).

A different nuance of the same rhetorical intent is found in Excerpt 2 (Table 2), where *Researcher A* explains to the participants a theoretical concept of the intervention model (the concept of *community* as a component of an activity system). *Researcher A* exemplifies such a concept by including the category “researchers in the room” (incumbent and junior researchers) as part of the *community* of the hospital activity system at that moment. In doing so, the researcher establishes a link between the practitioners and the researchers as “newcomers,” offering a new and broader perspective related to the theoretical concept presented.

Excerpt 3 (Table 2) shows another rhetorical use of the groups’ categorization. In this case, the identity marker used by the researcher refers to “we” as the board of directors, a category into which the researcher puts himself (“if we are the body”). In this excerpt, when talking about “body,” the participants are expressing two meanings interrelated: body and board of directors (*cuerpo* and *Cuerpo de Gobierno* in Spanish, respectively). In this regard, the researcher solicits a reflection on the multiple relationships of the board with other organizational actors through the metaphor of the “body” but also solicits the group participation by means of asking numerous questions to the participants. The researcher asks participants a question (“I ask you”) but formulates the content as if the participants had asked it (“if we are the body”). In this way, the researcher models a reflective attitude that consists of adopting the metaphor of the “body” and, in doing so, his rhetorical purpose is to support reflection and leave the participants the role of protagonists and those who have the main agency. The researcher builds such a discursive structure by means of questions and proposals, using a sort of “ventriloquism” (Carter, 2002), to encourage participants to question themselves on certain issues. At the same time, the researcher makes an effort to not be the only one to propose topics for discussion, which would be contrary to the formative intervention methodological principles. It is possible to affirm that the changing identity category of the researcher for a moment (“I ask you, if we are the body”) is one of the ways in which this nondirective and participatory approach is discursively performed, but at the same time, it reveals a commitment and an important rhetorical work aimed at negotiating the engagement of the participants.

In Excerpt 4 (Table 2), the researcher shows a different use of the pronoun “we” referring in this case to the category “all people in the room”. There is a shift from directing the question to board members to phrasing it using a broader and more inclusive identity category (“do you feel comfortable with that definition, or do we change it, modify it?”). Here *Researcher A* uses the category to activate a reflection that respects the bottom-up rule and he puts himself in the group, the group of those who seek a definition for the management activity of the board of directors. The attempt consists in constructing this enterprise as shared and collaborative, in this way the researcher accomplishes two things, i.e., he reaffirms the participatory approach and at the same time involves and orients the participants in such a defining task.

During the first session, the collective identity markers were used to create engagement and influence in the group without being directive, trying to respect the bottom-up participatory principle through questions and proposals, through the identity categories shown here. These identity markers are used by the researcher to promote reflection and action, but they do so by constructing the procedural directions as shared, as something that comes from the group of participants and not from the researchers. In doing so, the identity markers are employed to serve the purposes of the session and contribute to their achievement, serving mainly on *how* to achieve such purposes.

### Identity Categories Used by Researchers in the Fourth Session

The objectives of the fourth session consisted in supporting the participants to identify improvement areas and propose them alternatives and innovations conducive to the development of their work practices. In Excerpt 5 (Table 3), the identity category “incumbent researchers” is evoked to represent the researchers as the only ones with the competence to see what the object of the intervention is from a theoretical point of view (“the hospital as a whole, a collective activity”) (Engeström, 2015). These early “we’s” are in some ways a transgression from considering only the participants as repositories of knowledge, even though later the “we” of the researchers is evoked to empower the board reiterating the bottom-up participatory approach (“we want to put you in the leading role”). It seems that such an approach constrains the way researchers proceed discursively. In this regard, the use of such “we” pronouns by researchers performs a sort of compromise with respect to leaving everything completely open and in the hands of the participants. However, at the same time, the researchers tend to influence toward an outcome and move the intervention forward.

Excerpt 6 (Table 3) is still an example of the identity category “we” as “Incumbent researchers,” used in this case to support the participating group in identifying an improvement area and adopting a change proposal (“this is a model that we are proposing to you”). The rhetorical intentions linked to the category seem to continue with a negotiation process in order to launch resources conducive to change and, in doing so, stimulate participants’ transformative agency. However, the use of “we” as incumbent researchers achieves a distancing from the participants and connotes the researchers as holders of a methodological competence capable of orienting some aspects of the intervention. During the fourth session, such “we as researchers” seems less participatory than in the first session but exerts more pressure not surprisingly in such a central moment of the intervention.

In Excerpt 7 (Table 3), the researcher supports both participation and reflection by remarking on the board-of-directors category. On the other hand, the researcher puts himself as part of such an identity category to start a discussion about how the board members get involved with the local community. By considering himself as part of this group, the researcher opens up the discussion into the topic of patient inclusion, as proposed in the CL methodology (Virkkunen and Newnham, 2013) (“why not, bring a patient, let the patient tell us about a case, that we see from his point of view?”). Establishing a dialogue with the participants in such a direction seems to highlight a barrier observed by the researcher, who adopts this identity category to play a less intrusive role and support the participant group to overcome such barrier (“we call ‘breaking down the barriers’, crossing the barriers, the barrier between us and them”).

In Excerpts 6 and 7, we identified a passage from “we as researchers” (“this is a model that we are proposing to you”) to “we as board of directors” (why not, bring a patient … that we see from his point of view?). This change of identity category seems to push forward the adoption of the methodological principles that in the passage are constructed as shared and applied to the board. In Excerpt 8, the passage continues into the category “all the people in the room” that the researcher uses to support the reflection and discussion on the leadership style topic (“How to lead, as we would like than someone lead to us”).

In summary, during the fourth session, the identity markers are used to frame the focus of the intervention, propose resources conducive to change, and support the collective discussion according to the point of view of researchers. This latter rhetorical function (provide support for the collective discussion) results central to engaging the participants during the first and fourth sessions and can be considered as a specific action that allows the researchers to sustain the reflection process. In this way, researchers used such identity markers to develop the intervention and achieve its methodological goals.

## Discussion and Conclusion

In this study, we focused on how the researchers, among the various discursive devices at disposition, use the pronominal markers “we” to evoke social identities that are rhetorically functional to achieve the objectives of an intervention process carried out with a group of healthcare professionals.

In the data presented, researchers rely on the use of identity categories to engage participants in shared meaning making (Sacks, 1992), to negotiate their active involvement in the intervention, and to promote actions leading to change. Results show how the identity categories are strictly linked to the aims of the different sessions of the intervention. The identity categories marked by the researchers were used either as an affiliative device to create a closer engagement (“we” as “all the people in the room”) or to present the intervention as a shared and collaborative activity (“we” as “board of directors”) (in the first session) or rather as a distancing device between researchers and participants in order to assert knowledge of researchers and epistemic authority in finding proposals that lead to change (“we” as incumbent researchers) (in the fourth session).

We observed a tension connected to the identity categories used by researchers, consisting in the action of influencing, making proposals, and adopting an active role, as they try not to intervene too much and to respect the bottom-up participatory principle. This tension, in contrast, is just what qualifies and characterizes the “philosophy” at the base of the CL, as “guided activation of “self-managed” improvement processes” (Engeström et al., 1996; Virkkunen and Newnham, 2013).

The observed tension exemplifies a crucial aspect in relation to how the formative intervention model is discursively operationalized, particularly, in relation to how researchers seek to accomplish rhetorically its epistemological principles (Sannino and Engeström, 2017). The data presented contribute to shed light on an area of usefulness to analyze the formative intervention process and the contribution of researchers from an innovative approach. The empirical analysis of group membership and social identity markers has proved to be useful to assess emerging forms of collaboration in the context of the intervention and, more generally, as indicators of the effectiveness of professional practice of researchers in the formative intervention.

Future research developments plan to use such micro- and discursive analysis not only to investigate other contexts of formative intervention but also to analyze the use of social identity markers by group of participants as a “measure” of their involvement in the development of innovation in their work activity system.

## Data Availability Statement

The raw data supporting the conclusions of this article will be made available by the authors, without undue reservation.

## Ethics Statement

This study was reviewed and approved by Local Research and Ethics Committee, Mexican Institute of Social Security, approval number R-2017-1003-10. Written informed consent was obtained from all participants for their participation in this study.

## Author Contributions

HB provided the conception and design of the study, contributed to the data collection, analysis and interpretation of data, and drafting the article. FA and CZ provided the conception and design of the study, analysis and interpretation of data, drafting the article, and revised it critically for final submission. All authors have read and approved the manuscript.

## Conflict of Interest

The authors declare that the research was conducted in the absence of any commercial or financial relationships that could be construed as a potential conflict of interest.

## Publisher’s Note

All claims expressed in this article are solely those of the authors and do not necessarily represent those of their affiliated organizations, or those of the publisher, the editors and the reviewers. Any product that may be evaluated in this article, or claim that may be made by its manufacturer, is not guaranteed or endorsed by the publisher.
